# Application of Reaction Force Field Molecular Dynamics in Lithium Batteries

**DOI:** 10.3389/fchem.2020.634379

**Published:** 2021-01-13

**Authors:** Zhihao Shi, Jian Zhou, Runjie Li

**Affiliations:** Shagang School of Iron and Steel, Soochow University, Suzhou, China

**Keywords:** lithium battery, ReaxFF, molecular dynamics, simulation, SEI

## Abstract

Lithium batteries are widely used in portable electronic products. Although the performance of the batteries has been greatly improved in the past few decades, limited understanding of the working mechanisms at an atomic scale has become a major factor for further improvement. In the past 10 years, a reaction force field (ReaxFF) has been developed within the molecular dynamics framework. The ReaxFF has been demonstrated to correctly describe both physical processes and chemical reactions for a system significantly larger than the one simulated by quantum chemistry, and therefore in turn has been broadly applied in lithium batteries. In this article, we review the ReaxFF studies on the sulfur cathode, various anodes, and electrolytes of lithium batteries and put particular focus on the ability of the ReaxFF to reveal atomic-scale working mechanisms. A brief prospect is also given.

## Introduction

Owing to energy shortages, air pollution and population growth, intermittent energy sources (e.g., solar energy and wind power) have been utilized to substitute traditional ones, which greatly promotes the development of energy storage devices (Lv et al., 2018, 2019; Liu et al., 2020). Among these devices, due to high efficiency, zero-emission, and low noise, lithium batteries have attracted considerable attention and have already been used in various applications (Nishi, 2001; Goodenough and Kim, 2010). In the past few decades, energy density, rate capability, and cycling lifespan of lithium batteries have been significantly improved (Li et al., 2020; Sui et al., 2020; Wang et al., 2020b; Luo et al., 2021), but still do not fully meet the requirements of electric vehicles (Lu et al., 2013) and smart-grids. In order to further improve the performance of lithium batteries, a deeper understanding of the working mechanisms at an atomic scale, which to a large extent are unknown, is desired.

Molecular dynamics (MD) is a powerful atomic-scale simulation method and is suitable for studying processes such as interface reactions and microstructure evolution. MD exploits classical Newtonian functions to describe particle movement, in which interaction force between particles is calculated by potential equations/fields. To date, a large number of potential equations/fields have been developed for different systems. These equations/fields nevertheless can only describe physical interactions. Chemical reactions have to be handed over to another simulation method, i.e., quantum chemistry (QC), which however is computationally expensive and usually confined to small systems.

**Figure 1:**
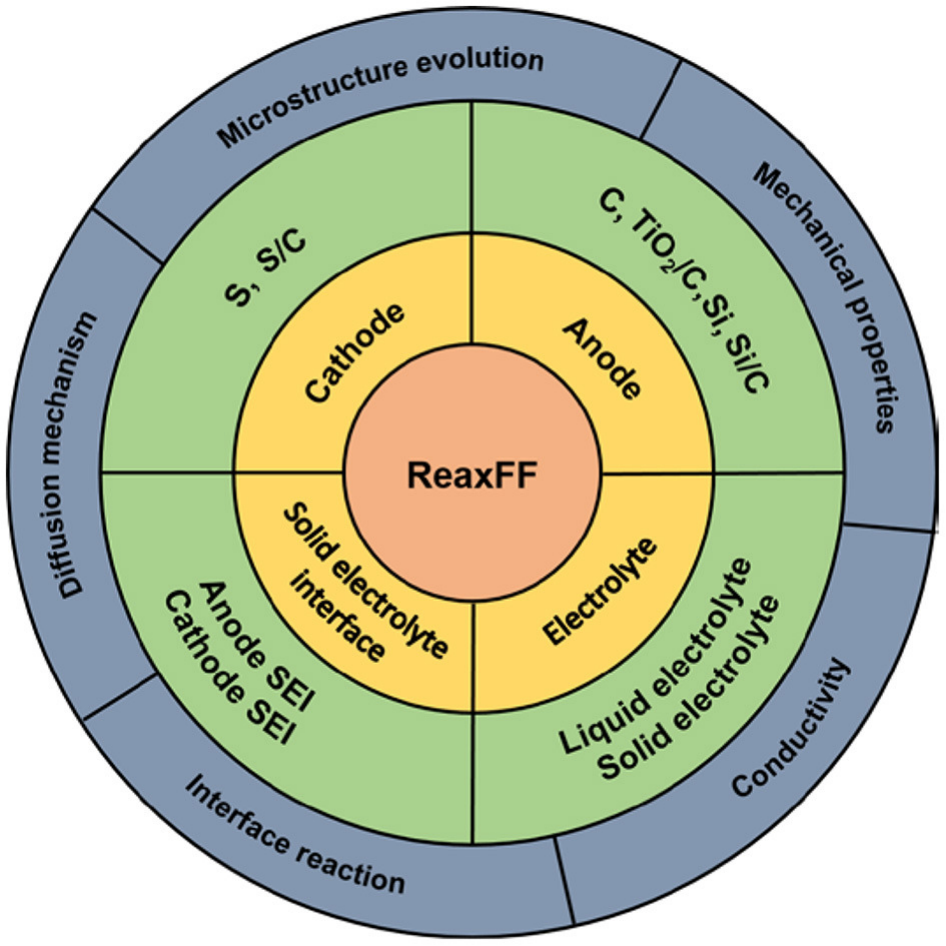
Overview of materials, properties, and mechanisms studied by ReaxFF.

In order to increase computational efficiency for large simulation systems, van Duin et al. (2001) proposed the reaction force field (ReaxFF) to simulate chemical reactions within the MD framework (hereinafter referred to as ReaxFF simulation). The ReaxFF simulation now covers most elements contained in cathode, anode, and electrolyte materials of lithium batteries. Based on the ReaxFF simulation, the sulfur cathode, various anodes, and electrolytes of lithium batteries have been investigated, as summarized in Figure 1. In this study, we review all these works by focusing on the ability of the ReaxFF to reveal atomic-scale working mechanisms of lithium batteries.

## Sulfur Cathode

Sulfur cathode is one of the most potential candidate materials for next-generation lithium batteries mainly due to is high theoretical specific capacity as 1675 mAh/g. Besides, sulfur is inexpensive and environmentally friendly. However, there are still many problems restricting commercialization of sulfur cathodes, such as huge volume expansion during cycling, poor electronic and ionic conductivities, and the polysulfide shuttle effect (Manthiram et al., 2013). In terms of simulation, the ReaxFF mainly focuses on the expansion behaviors of bulk sulfur, microstructure and composition evolution of sulfur nanoparticles, and interaction mechanisms between sulfur and carbon for a composite cathode.

Considering the expansion behavior and resulting change in mechanical properties of the sulfur cathode, Islam et al. (2015) simulated lithiation with different amounts of Li. The authors found that volume expansion rate and open circuit voltage curves obtained in their simulations were in good agreement with experimental findings, and both strength and fracture toughness of the cathode increased with the Li amount. Wang et al. (2017) further elucidated that this strengthening behavior resulted from a transition of the bonding network from non-bonded to covalently bonded atomic interactions. In addition, Wang et al. (2017) studied the expansion behavior under restriction conditions (suppressing in-plane expansion but allowing out-of-plane free expansion to occur). They found that the lithiation products of amorphous lithiated sulfur (a-Li_x_S) facilitated out-of-plane inelastic deformation at a low level of in-plane stress. This finding supports the optimization strategies of sulfur cathodes encapsulated by carbon nanotubes/nanofibers (Zheng et al., 2011).

In addition to the volume expansion, the lithium polysulfide species (e.g., Li_2_S_n_, n > 2) formed during lithium intercalation and deintercalation can also greatly affect battery performance. The microstructure and composition of Li_2_S nanoparticles during delithiation have been investigated by the ReaxFF. Li et al. (2018) compared the energy of four possible structures of Li_2_S_8_ nanoparticles, and concluded that the core-shell Li_2_S_8_ nanoparticles were most likely present during charging. Furthermore, via ring and fragment analyses with regard to Li_2_S and Li_2_S_8_ nanoparticles, it was found that a broad distribution of sulfur, lithium sulfide and lithium polysulfide species existed in both crystalline and amorphous Li_2_S and Li_2_S_8_ nanoparticles, which was attributed to surface atoms with significant strain, dangling bonds, and under-coordination.

Because of the ability to correctly describe intermolecular forces and Coulomb interactions, the ReaxFF has also been used to investigate interaction mechanisms between sulfur and carbon for a sulfur/graphene composite. Beltran and Balbuena (2018) studied a lithium insertion process for a mixture of 8-membered sulfur rings and graphene sheets. They found that the under-coordinated carbon atoms at the edge of the graphene could promote sulfur reduction, which caused the ring to directly reduce to short-chain sulfur, thus decreasing lithium polysulfide products. Ponce and Seminario (2020) compared the structural stability of the same composite undergoing fast and slow lithiation and found that the structure that experienced slow lithiation was more stable. These simulation results demonstrate that defects and lithiation rate can significantly affect the performance of the S/C composite.

## Anodes

Anodes are required to be of large specific capacity, high electronic/ionic conductivities and good cycle stability (Wang et al., 2020). At present, typical anode materials include carbon materials, TiO_2_, and silicon materials. Among them, TiO_2_ owns a theoretical specific capacity of 336 mAh/g (close to 372 mAh/g of graphite) and stable microstructure, but its conductivity is low (Wu et al., 2019). The theoretical specific capacity of Si is as high as 4200 mAh/g, but Si suffers serious volume expansion, which is usually alleviated by combining Si with other materials to form composite anodes (Reddy et al., 2013).

### Carbon Materials

Graphite and carbon nanotubes are considered to be good anode materials mainly due to their high electronic conductivity. The inevitable defects (such as vacancies, holes, and cracks) introduced during preparation however have a huge impact on these materials. The ReaxFF has been utilized to investigate defect effects on Li storage capacity, diffusion path of Li and mechanical properties of the anodes. In addition, structural stability of core-shell-structured multi-walled carbon nanotubes/TiO_2_ has also been studied.

The defects of a graphene sheet are thought to benefit Li storage. Raju et al. (2015) confirmed by the ReaxFF that vacancies can cause graphite to store more Li. Even with a low vacancy density of 2.1%, the maximum specific capacity of graphite was increased to 428 mAh/g. For defect-free graphite and carbon nanotubes, Li can only diffuse through the spaces between adjacent graphite layers and the center channels of carbon nanotubes, respectively. Raju et al. (2015) compared the diffusion barrier of Li across defect-free graphene and graphene with monovacancy/divacancy, and found that as vacancy number increased, the diffusion barrier decreased significantly, which indicate that the defect of vacancies provides Li with shortcut diffusion paths so as to increase charge and discharge rate.

Concerning mechanical properties, Huang et al. (2013) simulated tensile behaviors of carbon nanotubes with hole-like defects and single vacancy at different Li concentrations, and found that fracture strength decreased with an increase in Li concentration. The authors further revealed that, driven by the chemical potential difference, Li tended to accumulate around the defects and therefore weakened C-C bonds around the defects. As the defect number increased, this weakening effect became more significant due to more Li accumulation. A similar fracture mechanism was found in graphene by Yang et al. (2013).

Carbon nanotubes and TiO_2_ are combined to obtain core-shell multi-walled carbon nanotubes/TiO_2_ for good cycle performance (Yang et al., 2009). With the ReaxFF, the relationship between microstructure and performance was studied. Muñiz et al. (2016) calculated adsorption energy of TiO_2_ on the surface of multi-walled carbon nanotubes (MWCNT) and found that there was a strong electrostatic interaction between the two materials. The authors further focused on the thickness influence of the TiO_2_ shell on the microstructure, and found that at a specific thickness, MWCNT became highly symmetrical, meanwhile TiO_2_ became uniformly distributed. This optimal simulation microstructure agrees well with experimental observations (Cao et al., 2010).

### Silicon Materials

During lithiation, Li atoms diffuse into crystalline silicon (c-Si) anode and form amorphous lithiated silicon (a-Li_x_Si), which results in great volume expansion as well as a propagating interface between the two Si phases. In order to get insights into the effect of the volume expansion on the mechanical properties of the Si anode and atomic-scale mechanisms of both Li diffusion and Si phase transition around the interface, many ReaxFF works have been performed.

In terms of mechanical properties, Fan et al. (2013) calculated yield and fracture strength of a-Li_x_Si under various chemomechanical loading conditions. The authors found that changes in the mechanical properties were closely related to atomic bonding types. As Li concentration increased, the anode underwent plastic softening, which was caused by a decrease in Si-Si covalent bonds and increase in Li-Li metal bonds.

Considering interface reactions, Kim et al. (2014) and Ostadhossein et al. (2015) found that Li preferentially migrated along [110] direction in bulk c-Si, and the interface parallel to (111) plane propagated at the fastest speed. Jung et al. (2015) found that for c-Si nanowires, in addition to [110] direction, Li also preferentially migrated along [112] direction. Kim et al. (2014) examined composition around the interface and determined that on the two sides of the interface were c-LiSi and a-Li_15_Si_4_.

### Silicon Composite

SiO_2_ and Al_2_O_3_ have been used to coat Si for preventing volume expansion. These materials are thought to exhibit different mechanisms for Li diffusion into Si. Concerning SiO_2_ and Al_2_O_3_, Li reacts with them to form lithium silicon oxide and lithium aluminum oxide, respectively, and then diffuses across these products and Si. For carbonaceous layers, Li diffusion can only occur through defects and/or the spaces between adjacent graphite layers. For the different coating materials, many ReaxFF studies have been done.

The ReaxFF was utilized to elucidate the volume expansion inhibition effect as well as change of SiO_2_/Al_2_O_3_ mechanical properties during lithiation. Ostadhossein et al. (2016) studied the effect of lithiation on the mechanical properties of SiO_2_, and found that an increase in Li would lead to the enhancement of elastic softening and ductility of lithium silicon oxide compounds, which indicates that SiO_2_ can alleviate the mechanical deformation of Si. Jung et al. (2016) studied the structural evolution of core-shell-structured c-Si/a-SiO_2_ nanowires during lithiation, and suggested that the Li_x_Si formed from Si nanowires was under tensile stress, resulting in expansion behavior, while the one from SiO_2_/Si nanowires was subjected to compressive stress, thereby suppressing expansion. Kim et al. (2016) studied structural stability of Si with SiO_2_/Al_2_O_3_ shells of different thickness. It was found that the 4.5 Å Al_2_O_3_ shell was most stable, which was ascribed to the gradient of Young's modulus due to the Li concentration gradient. Based on the ReaxFF results, Kim et al. (2016) and Verners and Simone (2019) proposed to use a modulus gradient coating for optimizing Si anode. Recent experimental studies have shown that this strategy is effective (Jiao et al., 2020).

## Electrolytes

Electrolytes, as a carrier for lithium ion transport, need to be of high ionic conductivity, thermal stability, and non-reaction with electrodes (Wang et al., 2020a). The most used electrolyte for lithium batteries is high dielectric constant ethylene carbonate (EC) mixed with low viscosity chain carbonate, e.g., dimethyl carbonate (DMC), ethyl methyl carbonate (EMC), and diethyl carbonate (DEC), or carboxylate acid ester, e.g., methyl acetate (MA) (Xu, 2004). The ReaxFF has been successfully applied to investigate thermal stability and ionic conductivity of the electrolytes.

For the liquid electrolytes, high-temperature degradation reactions were studied. Gao and Lu (2019) investigated the influence of solvent type, LiPF_6_ salt concentration, and temperature on thermal stability of three electrolytes. It was found that DMC possessed the highest thermal stability, followed by EMC and DEC. In addition, the gas phase products of pyrolysis of these three electrolytes all contained CO_2_, H_2_, CH_4_, C_2_H_4_, C_2_H_6_, and CH_3_CH_2_F. These results are consistent with the experimental findings. The authors further studied the thermal degradation mechanism of the three electrolytes and found that during the decomposition process, ${\text{PF}}_{6}^{-}$ ions tended to preferentially form PF_5_ and F^−^ ions. The PF_5_ lewis acid promotes the subsequent degradation reactions of different electrolytes by attacking C-O bonds.

## Solid Electrolyte Interface

During the first cycle of a lithium battery, the electrolyte usually reacts with the electrode to form a solid electrolyte interface (SEI) film. On one hand, the SEI film consumes active lithium and electrolyte, resulting in capacity degradation, increased battery internal resistance and reduced energy density. On the other hand, the film improves cycle stability and prevents further decomposition of electrolyte. The formation mechanisms of nanometer-thick SEI film has not been fully understood (Wang et al., 2018). Through the ReaxFF, composition of the SEI film and reaction mechanisms have been predicted.

The ReaxFF was shown to be able to simulate the formation process of the SEI film. In terms of a graphite anode, Kim et al. (2011) and Bertolini and Balbuena (2018) simulated the reaction process of Li and common electrolytes. Kim and coauthors found that the SEI film separated into an organic layer and an inorganic layer according to product composition. According to the Li distribution, Bertolini and Balbuena determined that the SEI film contained a dense phase and a porous phase. The latter was further found to be composed of a nest phase and a dispersed phase based on connectivity of Li. SEI film was also determined for cathodes. Reddivari et al. (2017) studied a reaction between LiMn_2_O_4_ and a mixed electrolyte. The authors found that the SEI film on the cathode was mainly composed of aldehydes, esters, and alcohol organic compounds, but there was no inorganic carbonate or organic lithium salt.

Regarding reaction mechanisms, Kim et al. (2011) proposed two decomposition mechanisms for reaction of DMC and EC on Li anode. Bedrov et al. (2012) revealed a competitive reaction mechanism for reaction of EC on Li anode. Most of the decomposition products are in good agreement with experimental results, while discrepancies possibly arose from deviation of Li concentration at the interface or electron injection speed.

## Conclusions and Outlook

To summarize, based on the various works mentioned above, the ReaxFF has been proved to be a powerful tool for investigating lithium battery systems. The atomic-scale mechanisms revealed by the ReaxFF is expected to be useful for optimizing battery structures and also improving battery performance.

In the future, the ReaxFF simulation can be utilized for many other processes, such as reactions between Li and trace water in electrolyte, diffusion of polysulfide in electrolyte and dendrite growth at electrolyte-anode interface. ReaxFF can further discover the role of composite electrode materials and functional coatings in these processes. In particular, the ReaxFF simulation is readily extended to sodium-ion, potassium-ion and lithium-air battery systems, which are popular candidates for next-generation batteries.

## Author Contributions

All authors collected and summarized literature and wrote the manuscript. JZ provided guidance and revised the manuscript.

## Conflict of Interest

The authors declare that the research was conducted in the absence of any commercial or financial relationships that could be construed as a potential conflict of interest.
